# Expression of Components of the Renin-Angiotensin System by Cancer Stem Cells in Renal Clear Cell Carcinoma

**DOI:** 10.3390/biom11040537

**Published:** 2021-04-07

**Authors:** Sam Siljee, Bridget Milne, Helen D. Brasch, Nicholas Bockett, Josie Patel, Paul F. Davis, Andrew Kennedy-Smith, Tinte Itinteang, Swee T. Tan

**Affiliations:** 1 Gillies McIndoe Research Institute, Wellington 6242, New Zealand; sam.siljee@gmri.org.nz (S.S.); bridget.milne@icloud.com (B.M.); hdquinn@fastmail.fm (H.D.B.); nick.bockett@gmri.org.nz (N.B.); josie.patel@gmri.org.nz (J.P.); paul.davis@gmri.org.nz (P.F.D.); tinte01@yahoo.com (T.I.); 2 Department of Urology, Wellington Regional Hospital, Wellington 6021, New Zealand; aks@xtra.co.nz; 3 Wellington Regional Plastic, Maxillofacial and Burns Unit, Hutt Hospital, Lower Hutt 5010, New Zealand; 4 Department of Surgery, The Royal Melbourne Hospital, The University of Melbourne, Melbourne, VIC 3010, Australia

**Keywords:** renal clear cell carcinoma, renin-angiotensin system, cancer stem cells, renin, pro-renin receptor, angiotensin-converting enzyme, angiotensin-converting enzyme 2, angiotensin II receptor 1, angiotensin II receptor 2

## Abstract

This study investigated the expression of components of the renin-angiotensin system (RAS) by cancer stem cells (CSCs) we have recently demonstrated in renal clear cell carcinoma (RCCC). Fifteen RCCC tissue samples underwent immunohistochemical staining for components of the RAS: renin, pro-renin receptor (PRR), angiotensin-converting enzyme (ACE), angiotensin-converting enzyme 2 (ACE2), and angiotensin II receptor 2 (AT_2_R). Immunofluorescence co-staining or double immunohistochemical staining of these components of the RAS with stemness-associated markers OCT4 or KLF4 was performed on two of the samples. Protein and transcript expression of these components of the RAS in six RCCC tissue samples was investigated using western blotting and reverse transcription quantitative polymerase chain reaction (RT-qPCR), respectively. In addition, angiotensin II receptor 1 (AT_1_R) was investigated using RT-qPCR only. Immunohistochemical staining demonstrated expression of renin, PRR, and ACE2 in 11, 13, and 13 out of 15 RCCC samples, respectively, while AT_2_R was expressed in all 15 samples. ACE was detected in the endothelium of normal vasculature only. Double immunohistochemical staining demonstrated localization of ACE2, but not renin, to the KLF4+ CSCs. Immunofluorescence staining showed localization of PRR and AT_2_R to the OCT4+ CSCs. Western blotting confirmed protein expression of all components of the RAS except renin. RT-qPCR demonstrated transcript expression of all components of the RAS including AT_1_R, but not AT_2_R, in all six RCCC tissue samples. This study demonstrated expression of PRR, ACE2, and AT_2_R by the CSCs within RCCC. Further studies may lead to novel therapeutic targeting of CSCs by manipulation of the RAS in the treatment of this aggressive cancer.

## 1. Introduction

Renal cell carcinoma (RCC) is the 9th most common cancer worldwide, with renal clear cell carcinoma (RCCC) being the most common, contributing to approximately 70% of all RCCs [1]. RCCC arises from the epithelial cells lining the proximal convoluted tubules of the kidney [2]. It presents with a variety of symptoms, but is often asymptomatic in early stages [1,3]. This is significant in the light of stage-dependent prognosis, with the 5-year survival rate decreasing from 91.7% for localized disease, to 12.3% for metastatic disease [4]. The incidence of RCCC increases with age, and is highest in developed countries due to incidental diagnosis secondary to the availability of imaging [1,5]. The current understanding of the pathogenesis of RCCC relates to both genetics and environmental exposure [4]. Deletions in the short arm of chromosome 3 occur in 95% of RCCC [6]. Specifically, this involves loss of the VHL gene, the normal product of which is a tumor suppressor protein [6,7]. Loss of the VHL gene translates to increased cellular growth factors which in turn facilitates cancer development [6,7]. Known risk factors for RCCC include smoking, obesity and hypertension [8].

Treatment for RCCC depends on disease stage, localized disease may be cured by partial or total nephrectomy. There is minimal role for traditional chemotherapy and radiotherapy. Targeted systemic therapies such as tyrosine kinase inhibitors or immunotherapy may be used for advanced disease [3,9]. However, disease response is variable and relatively unpredictable; survival of patients with metastatic RCCC remains poor [4].

Cancer stem cells (CSCs), the proposed origins of cancer, possess self-renewal and multi-potent potential, and drive tumor growth [10]. CSCs have been previously demonstrated in RCCC [5,11,12,13,14], and are responsible for radiotherapy and chemotherapy resistance of RCCC [3]. We have recently demonstrated subpopulations of CSCs in RCCC expressing the transcription factors NANOG, OCT4, SOX2, KLF4, and c-MYC [5]—stemness-associated markers involved in the generation of induced pluripotent stem cells (iPSCs) [15,16]. The expression of these markers has been shown to confer worse prognosis in RCCC [17].

The renin-angiotensin system (RAS) classically regulates blood pressure and body fluid homeostasis [18]. We have demonstrated the expression of components of the RAS by CSCs in many cancer types including glioblastoma [19], oral cavity squamous cell carcinoma of different subsites [20,21,22], primary head and neck cutaneous squamous cell carcinoma (HNcSCC) [23] and metastatic HNcSCC (mHNcSCC) [24], metastatic malignant melanoma (MM) to the brain [25] and regional lymph nodes [26], and metastatic colon adenocarcinoma [27]. We have also demonstrated expression of cathepsins B, D and G which constitute bypass loops of the RAS [28], in a number of cancer types [29,30,31,32]. This underscores the critical role of the RAS in the development and progression of cancer [28,33].

Components of the RAS, i.e., renin, pro-renin receptor (PRR), angiotensin-converting enzyme (ACE), ACE2, angiotensin II receptor 1 (AT_1_R), and angiotensin II receptor 2 (AT_2_R), have been associated with the development of cancer [28,33]. Renin is activated from its inactive form, pro-renin by PRR, and converts angiotensinogen to angiotensin I (ATI). Renin has been implicated in carcinogenesis through Wnt/β-catenin signaling [28,34], with PRR over-expression also being linked to increased cellular proliferation [28,35]. Additionally, initial PRR over-expression has been linked to early stages of tumorigenesis [28,35]. ACE further converts ATI to angiotensin II (ATII), both of which act on AT_1_R and AT_2_R. ACE inhibitors (ACEIs) confer a protective effect against cancer [33,36,37]. ACE2 counteracts the action of ACE, by cleaving ATII downstream to form angiotensin (1–7) [38] which has anti-angiogenic and anti-metastatic actions, in addition to reducing endothelial-to-mesenchymal transition [39,40,41]. Over-expression of AT_1_R is associated with tumor invasiveness [36]. AT_2_R generally attenuates the effects of AT_1_R and consequently has a protective role against cancer development [40,42].

There is increasing evidence showing that RAS inhibitors (RASIs), specifically ACEIs and AT_1_R blockers (ARBs), significantly improve the overall survival of patients with RCCC, and enhance the efficacy of vascular endothelial growth factor targeted therapies [43,44,45,46,47,48,49]. A recent meta-analysis demonstrates survival benefits for both ACEIs and ARBs in RCC [37].

This study investigated the expression of components of the RAS: renin, PRR, ACE, ACE2, AT_1_R, and AT_2_R in relation to the CSC subpopulations which we have recently identified in RCCC [5], using immunohistochemical and immunofluorescence staining, western blotting (WB), and reverse transcription quantitative polymerase chain reaction (RT-qPCR).

## 2. Materials and Methods

### 2.1. RCCC Tissue Samples

RCCC tissue samples from eight female and seven male patients, aged 37–88 (mean 66.6) years, including those used in our previous study [5] (Table S1), were sourced from the Gillies McIndoe Research Institute Tissue Bank. This study was approved by the Northern B Health and Disability Ethics Committee (Ref. 16/NTB/10) with written informed consent from all participants.

### 2.2. Histology and Immunohistochemical Staining

Hematoxylin and eosin (H&E) staining was performed on 4 µm-thick formalin-fixed paraffin-embedded consecutive sections of the 15 RCCC tissue samples to confirm the presence of the tumor on the slides. Immunohistochemical staining of sections of RCCC was then performed on the Leica BOND^TM^ RX auto-stainer (Leica, Nussloch, Germany) using primary antibodies for renin (1:500; cat#14291-1-AP, Proteintech, Rosemont, IL, USA), PRR (1:500; cat#ab40790, Abcam, Cambridge, MA, USA), ACE (1:30; cat#ab11734, Abcam), ACE2 (1:200; cat#MAB933 R&D Systems, Minneapolis, MN, USA), and AT_2_R (1:2000; cat#NBP1-77368, Novus Biologicals, Littleton, CO, USA) with 3,3′-diaminobenzidine as the chromogen. Immunohistochemical staining was completed using the BOND polymer refine detection kit (cat#DS9800, Leica).

For the co-localization of renin and ACE2 to CSCs, double immunohistochemical staining was undertaken using the same antibody and concentration as above, with KLF4 (1:100; cat#AF3640, R&D Systems), using 3,3′-diaminobenzidine as the chromogen alongside the BOND Polymer Refine Red Detection kit (ready-to-use, cat#DS9390, Leica). KLF4 combinations used a rabbit anti-goat linker antibody (1:500; cat#305-005-045, Jackson ImmunoResearch Laboratories, West Grove, PA, USA). Double immunohistochemical staining was used to co-localize ACE2 as this antibody was not optimized for immunofluorescence staining.

Human tissues used for positive controls in immunohistochemical staining were placenta for PRR, and normal kidney for renin, ACE, ACE2, and AT_2_R. Human tissue negative controls used were salivary gland for renin and AT_2_R, colon for PRR, and skin for ACE and ACE2. Isotype negative controls were prepared on sections of RCCC tissue samples using a primary isotype rabbit antibody (ready-to-use, cat#IR600, Dako, Glostrup, Denmark) for renin, PRR, and AT_2_R, isotype mouse antibody (ready-to-use, cat#IR750, Dako) for ACE and ACE2, or isotype goat antibody (1:250, cat#02-6202, Invitrogen, Carlsbad, CA, USA) for KLF4. Immunohistochemical staining was not performed for AT_1_R due to a lack of specific antibodies [50,51,52,53].

### 2.3. Immunofluorescence Staining

Immunofluorescence staining was performed on sections of two RCCC samples from the original cohort of 15 patients. Localization of the components of the RAS in relation to the CSCs we have previously identified [5] was achieved by co-staining with the stemness-associated marker OCT4. The primary antibodies and concentrations used for detection of the components of the RAS were identical to those used for immunohistochemical staining, in addition to OCT4 (1:30; cat#309M-16, Cell Marque, Rocklin, CA, USA). Immunofluorescence staining was completed using VectaFluor Excel anti-mouse DyLight 488 (ready-to-use; cat#DK-2488, Vector Laboratories, Burlingame, CA, USA) and Alexa Fluor anti-rabbit 594 (1:500; cat# A21207, Invitrogen). Isotype negative controls were prepared as described for immunohistochemical staining.

### 2.4. Image Analysis

Immunohistochemical-stained slides were visualized and imaged using the Olympus BX53 light microscope, fitted with an Olympus SC100 digital camera (Olympus, Tokyo, Japan), and processed with cellSens 2.0 software (Olympus). Immunofluorescence-stained slides were viewed and imaged with the Olympus FV1200 biological confocal laser-scanning microscope and processed with cellSens Dimension 1.11 (Olympus).

### 2.5. RT-qPCR

Total RNA was isolated from six available snap-frozen RCCC tissue samples of the original cohort of 15 patients. From each sample, approximately 20 mg of snap-frozen tissue was homogenized using the Omni Tissue Homogenizer (Omni International, Kennesaw, GA, USA). Total RNA was then extracted using the RNeasy Mini kit (cat#74104, Qiagen, Hilden, Germany) according to the manufacturer’s instructions. An on-column DNase digest (cat#79254, Qiagen) step was included. RNA was quantified using a NanoDrop 2000 Spectrophotometer (Thermo Fisher Scientific, Waltham, MA, USA). Transcript expression was analyzed in triplicate using the Rotor-Gene Q (Qiagen), Rotor-Gene Multiplex RT-PCR Kit (cat#204974, Qiagen), and TaqMan Gene Expression Assay primer probes (cat#4331182, Thermo Fisher Scientific) on 40 ng of RNA. The TaqMan primer probes used were renin (Hs00982555_m1), PRR (Hs00997145_m1), ACE (Hs00174179_m1), ACE2 (Hs01085333_m1), AT_1_R (Hs00258938_m1), and AT_2_R (Hs00169126_m1) (cat#4331182, Thermo Fisher Scientific). Gene expression was normalized to the reference genes GAPDH (Hs99999905_m1) and PUM1 (Hs00206469_m1) (cat#4331182, Thermo Fisher Scientific). Universal human reference RNA (UHR; cat#CLT636690, Takara, Shiga, Japan)—total RNA from a range of healthy human adult tissues—was used as the calibrator for the 2^∆∆Ct^ analysis. Nuclease-free water was run as the no template control, and RNA from PC3 cells (renin), uterine fibroid tissue (PRR, ACE, AT_1_R, and AT_2_R), or HepG2 cells (ACE2) were used as positive controls. End-point amplification product specificity was confirmed with 2% agarose gel (cat#G402002, Thermo Fisher Scientific) electrophoresis and imaged using the ChemiDoc MP (Bio-Rad, Hercules, CA, USA) and Image Lab 6.0 software (Bio-Rad). Graphs were generated using GraphPad Prism (v8.0.2, San Diego, CA, USA) and results expressed as fold-change relative to UHR. A biologically significant fold-change cut off was set at 2.0 for up-regulated and 0.5 for down-regulated genes.

### 2.6. Western Blotting

Total protein, from the same six snap-frozen RCCC samples used for RT-qPCR, was extracted by pestle homogenization (cat#PES-15-B-SI, Corning, Tewsksbury, MA, USA) in ice-cold Radioimmunoprecipitation assay buffer (cat#89900, Pierce Biotechnology, Rockford, IL, USA) supplemented with a protease and phosphatase inhibitor cocktail (cat#78440, Pierce Biotechnology). Protein was quantified using a BCA assay (cat#23227, Pierce Biotechnology), and diluted in an equal volume of 2× LDS (cat#B0007, Invitrogen). Protein was separated by one-dimensional sodium dodecyl sulfate polyacrylamide gel electrophoresis (20 µg total protein per sample), prior to being transferred to polyvinylidene difluoride membranes (cat#IB24001, Invitrogen). Protein detection was performed on the iBind flex (cat#SLF2000, Thermo Fisher Scientific) using primary antibodies for PRR (1:250; cat#ab40790, Abcam), ACE (1:200; cat#sc-12184, Santa Cruz Biotechnology, Dallas, TX, USA), ACE2 (1:500, cat#MAB933, R&D Systems), AT_2_R (1:500; cat#ab92445, Abcam), and α-tubulin (1:2000; cat#62204, Invitrogen). Appropriate secondary antibodies were goat anti-rabbit horse radish peroxidase (HRP) conjugate (1:1000, cat#111-035-045, Jackson ImmunoResearch Laboratories) for PRR, goat anti-mouse HRP (1:2000; cat#ab6789, Abcam) for ACE and ACE2, donkey anti-rabbit HRP (1:1000; cat#SA1-200, Thermo Fisher Scientific) for AT_2_R, and donkey anti-mouse Alexa Fluor 488 (1:1000, cat#A21202, Invitrogen) for α-tubulin. Clarity Western ECL (cat#1705061, Bio-Rad) was used as the substrate for visualizing HRP probed protein bands and the ChemiDoc MP Imaging System (Bio-Rad) and Image Lab 6.0 software (Bio-Rad) were used for band detection and analysis. Positive controls were human tonsil for PRR, mouse lung for ACE, human kidney for ACE2, and HepG2 cell line for AT_2_R. WB for renin was abandoned after multiple antibodies failed to produce a single specific band.

## 3. Results

### 3.1. Renin, ACE2, PRR and AT_2_R Were Expressed in RCCC Tissue Samples

**Figure 1:**
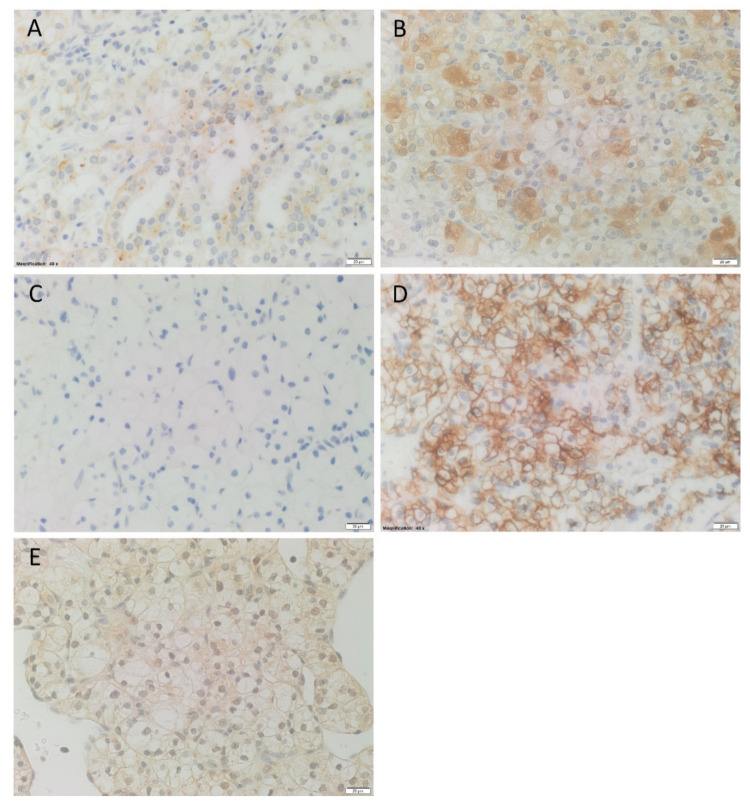
Representative immunohistochemical-stained images of renal clear cell carcinoma tissue samples. Renin (**A**, brown) and PRR (**B**, brown) showed cytoplasmic staining of the tumor cells. ACE (**C**, brown) was not present within the tumor. ACE2 (**D**, brown) showed mostly membranous, with some cytoplasmic, staining of the tumor cells. AT_2_R (**E**, brown) was expressed in the cytoplasm and nucleus of the cells within the tumor. Nuclei were counterstained with hematoxylin (**A**–**E**, blue). Original magnification: 400×. Scale bar: 20 µm.

H&E staining confirmed the presence of RCCC for all 15 tissue samples (Figure S1A). Patient demographic details are summarized in Table 1, with additional details presented in Table S1. Immunohistochemical staining demonstrated weak to moderate cytoplasmic staining of renin in 11 RCCC samples (Figure 1A). PRR showed cytoplasmic expression in 13 cases, with variable strength of staining (Figure 1B). ACE was present on the endothelium of normal vessels (Figure S1B) in all samples but was not present within the tumor (Figure 1C) in all 15 samples. ACE2 demonstrated a heterogenous membranous staining in 13 cases, with granular cytoplasmic staining in eight cases (Figure 1D). The staining pattern for AT_2_R demonstrated cytoplasmic staining of tumor cells in all samples, and variable nuclear staining of 13 cases (Figure 1E). Results of immunohistochemical staining are summarized in Table S3.

Positive human control tissues demonstrated the expected staining pattern for renin (Figure S1C) in normal kidney; PRR (Figure S1D) in placenta; and ACE (Figure S1E), ACE2 (Figure S1F), and AT_2_R (Figure S1G) in normal kidney. No staining was present in the isotype negative control (Figure S1H), or tissue negative controls (Figure S2A–E).

### 3.2. ACE2, PRR and AT_2_R but Not Renin Were Expressed by CSCs in RCCC Tissue Samples

**Figure 2:**
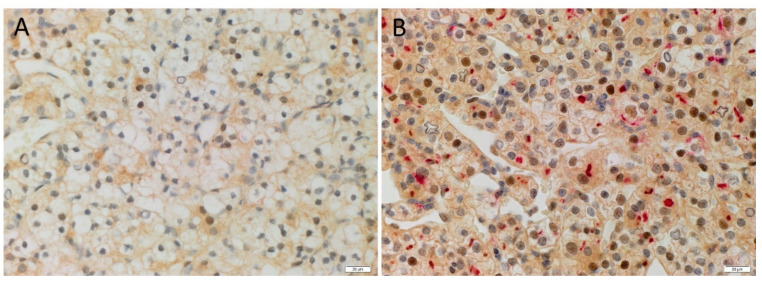
Representative double immunohistochemical stained images demonstrating no expression of renin (**A**, red) and expression of ACE2 (**B**, red) on the KLF4+ (**A**,**B**, brown) CSCs. Nuclei were counterstained with hematoxylin (**A**,**B**, blue). Original magnification: 400×. Scale bar: 20 µm.

Double immunohistochemical staining did not show renin (Figure 2A, red) on the KLF4+ (Figure 2A, brown) CSCs in the RCCC tissue samples. ACE2 (Figure 2B, red) was expressed by the KLF4+ (Figure 2B, brown) CSCs. Positive controls for double immunohistochemical staining showed appropriate cytoplasmic staining for renin (Figure S3A, red) in the bronchus, ACE2 (Figure S3B, red) in the kidney, with nuclear staining of KLF4 (Figure S3C, brown) in the colon epithelium. There was no staining on the isotype negative controls in the bronchus (Figure S3D), the kidney (Figure S3E), or the colon (Figure S3F).

**Figure 3:**
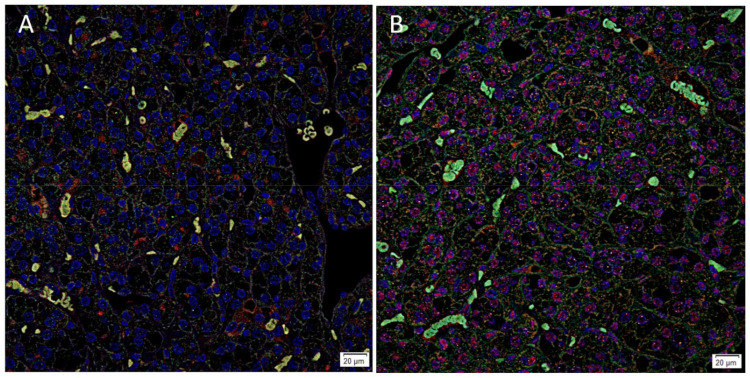
Representative immunofluorescence-stained sections of renal clear cell carcinoma tissue stained for of AT_2_R (**A**, red) with OCT4 (**A**, green). AT_2_R (**B**, red) was detected on the OCT4+ (**B**, green) CSCs. Cell nuclei were counterstained with 4′,6-diamidino-2-phenylindole (**A**,**B**, blue). Original magnification 400×. Scale bar: 20 µm.

Immunofluorescence staining showed cytoplasmic expression of PRR (Figure 3A, red) on the OCT4+ (Figure 3A, green) CSCs and AT_2_R (Figure 3B, red) predominantly expressed within the nuclei of the OCT4+ (Figure 3B, green) CSCs in the RCCC tissue samples. Split images of immunofluorescence staining presented in Figure 3 are shown in Figure S4. The negative controls demonstrated minimal staining (Figure S4E).

### 3.3. Renin, PRR, ACE, ACE2, and AT_1_R Transcripts Were Expressed in RCCC Tissue Samples

**Figure 4:**
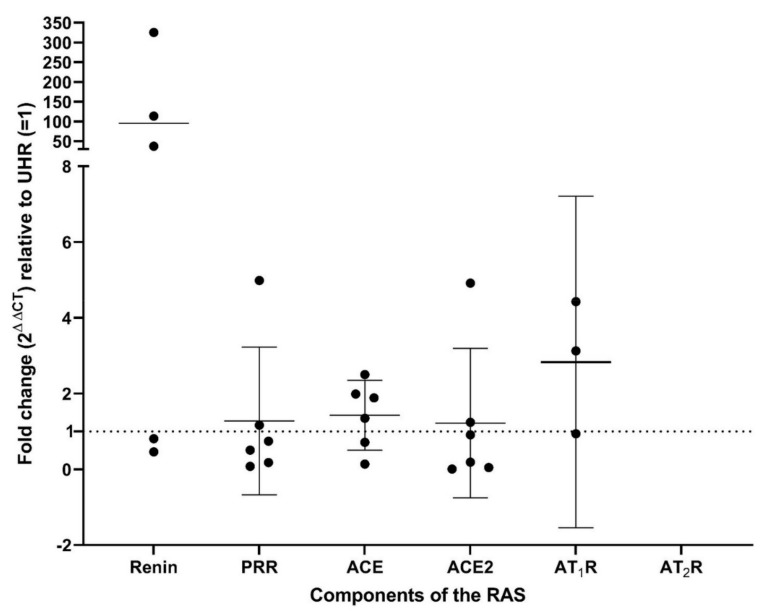
Fold-change (2^ΔΔCT^) in gene expression of the components of the renin-angiotensin system (RAS): renin, PRR, ACE, ACE2, AT_1_R, and AT_2_R, determined by RT-qPCR on total RNA extracted from six renal clear cell carcinoma tissue samples. CT values were normalized to the reference genes GAPDH and PUM1 and displayed as expression relative to universal human reference RNA (UHR). Error bars represent 95% confidence intervals of the mean.

Expression of PRR, ACE, ACE2, and AT_1_R was detected by RT-qPCR, at levels similar to healthy UHR, with renin detected at increased levels (Figure 4). Renin was detected in five of the six RCCC tissue samples, with expression split into two groups. Two samples showed expression comparable to that of healthy UHR, while the other three were highly up-regulated relative to healthy UHR. AT_1_R was detected in only three of the samples. AT_2_R was detected in healthy UHR, but not in any of the RCCC samples. Fold-change values are presented in Table S2, and specific amplification was confirmed by gel electrophoresis of PCR products (Figure S5). The expected sized amplicons were observed, with no products visible in the no template control lanes. Subset analysis of PCR data is presented in Figure S6. Statistical significance determined by un-paired t-test showed no significant differences found with gender, age at diagnosis, or tumor grade. Tumor stage showed significantly increased ACE2 expression at stage T2a, however, there was no significant difference with any of the other markers.

### 3.4. Western Blotting Confirmed the Presence of PRR, ACE, ACE2 and AT_2_R Proteins in RCCC Tissue Samples

**Figure 5:**
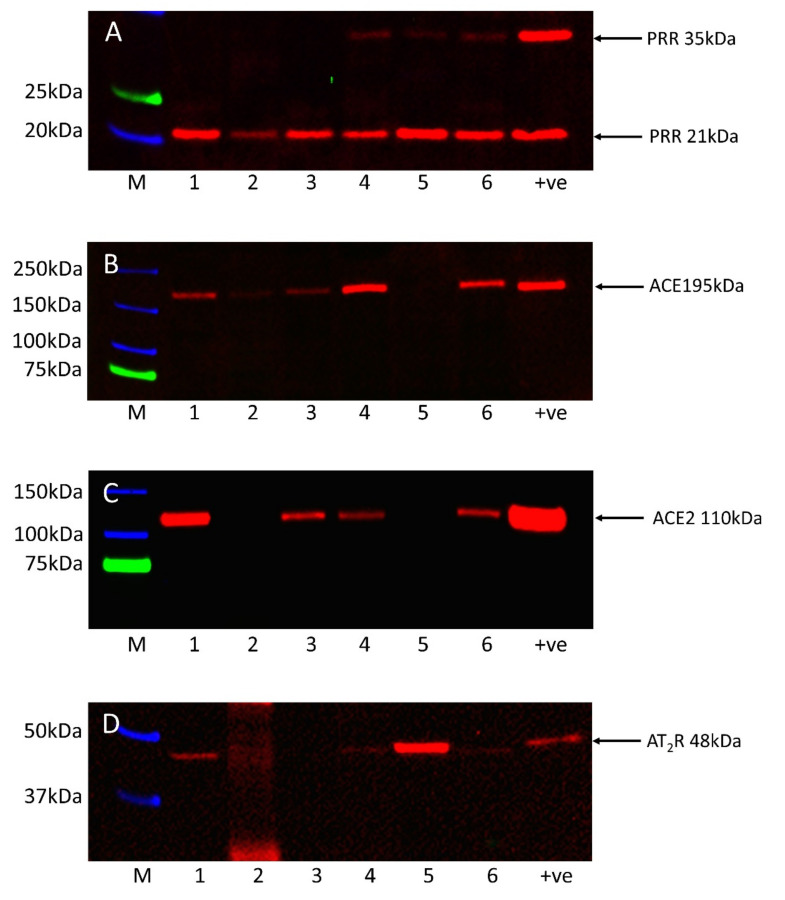
Representative cropped images of western blot analysis of total protein extracted from six renal clear cell carcinoma tissue samples demonstrating the presence of PRR (**A**, red), ACE (**B**, red), ACE2 (**C**, red), and AT_2_R (**D**, red). Full-length blots are presented in Figure S7.

WB performed on the six snap-frozen RCCC samples demonstrated bands at the expected molecular weights for PRR, ACE, ACE2 and AT_2_R (Figure 5). PRR was present in all six samples with the soluble 21 kDa form detected in all six samples and the full-length 35 kDa transmembrane isoform detected in three of the six samples (Figure 5A, red). ACE was detected at the appropriate molecular weight of 195 kDa in five of the six samples (Figure 5B, red). ACE2 was detected at the expected molecular weight of 110 kDa in four of six samples (Figure 5C, red). AT_2_R was detected at the molecular weight of approximately 48 kDa in four of the six samples (Figure 5D, red). Blotting for renin with various antibodies failed to produce a single specific band. WB results are summarized in Table S2. Full-length images of all blots are available in Figure S7A–D. α-Tubulin confirmed similar total protein loading for each sample (Figure S6E, red). Rabbit IgG isotype controls confirmed an instance of nonspecific staining on the blot for AT_2_R (Figure S6F, red).

## 4. Discussion

This study demonstrated the expression of five components of the RAS: renin, PRR, ACE2, AT_1_R, and AT_2_R in RCCC with PRR, ACE2, and AT_2_R localized to the CSCs in RCCC which express the transcription factors OCT4, NANOG, SOX2, KLF4, and c-MYC [5]. These results contribute to the growing evidence linking the RAS to carcinogenesis. Immunohistochemical staining showed that AT_2_R was expressed in all 15 RCCC tissue samples examined, with renin, PRR, and ACE2 expressed in the majority of samples. The detection of PRR and ACE2 by immunohistochemical staining was confirmed by WB and RT-qPCR performed on six of the RCCC samples. WB confirmed expression of AT_2_R in four samples, however, RT-qPCR did not show expression of AT_2_R. WB was not available for renin, but its expression was confirmed by RT-qPCR. It is interesting that all of the three patients with up-regulated renin mRNA were female, with the others being male. However, this difference was not statistically significant (*p* = 0.2074) (Welch two sample t-test, R version 4.0.3). Further subset analysis of the RT-qPCR data was also non-significant (Figure S6). The other components of the RAS were detected at levels similar to UHR, however, the significance of this relative expression is difficult to interpret as UHR may not necessarily reflect expression levels in normal kidney. Expected WB bands for PRR include the transmembrane form at 35 kDa and the soluble isoform at approximately 28 kDa [28]. As a 21 kDa band was not detected in either the negative control or the rabbit IgG isotype control (Figure S5F), the 21 kDa band detected by WB suggests the presence of a degraded form of the PRR protein. Detection of ACE by WB and RT-qPCR likely reflects the normal vasculature as seen in immunohistochemical staining, rather than expression by the tumor itself. Those samples which expressed ACE, as demonstrated by WB, also expressed ACE2. The co-expression of these two components of the RAS has been noted previously [54]. AT_2_R was detected at 48 kDa by WB which is larger than the theoretical size of 41 kDa, suggesting the presence of a glycosylated form [22], with additional non-specific banding as confirmed by the isotype control (Figure S5F).

Double immunohistochemical staining demonstrated expression of ACE2 by the KLF4+ CSCs. Immunofluorescence co-staining demonstrated localization of PRR and AT_2_R to the OCT4+ CSCs we have previously identified in RCCC [5]. However, expression of renin was not demonstrated by double immunohistochemical staining. This could be due to overstaining of the KLF4 which might have masked the weak renin staining shown on single immunohistochemical staining. Alternatively, this may be due to antibody interaction relative to single immunohistochemical staining. Due to a lack of specific antibodies for AT_1_R [50,51,52,53], we were unable to demonstrate its protein expression and localization to the CSCs in this tumor.

Renin is a secreted protein with an isoform lacking exon 1 which encodes for a non-secreted form [55]. The membranous isoform has a function distinct from secreted renin, and has been demonstrated to protect cells from necrotic death [56], suggesting a local effect of this non-secreted renin in RCCC.

PRR has not been studied in-depth in the context of RCCC. However, it is known to play a role in renal injury and fibrosis as a critical element in Wnt/β-catenin signaling [57], which is a known pathway in renal carcinogenesis [58,59,60].

We did not detect ACE within the tumor; however, it was detected in surrounding normal vasculature, consistent with other work [61,62]. This suggests a loss of function of ACE in RCCC, however, this does not preclude local conversion of ATI to ATII. There are enzymes such as chymase that constitute bypass loops of the RAS [63] which could catalyze this conversion. Chymase has been demonstrated in other cancer types including gastric [64], lung [65], and uterine cervical [66] carcinoma.

The finding of ACE2 expression in RCCC is consistent with work by Errarte et al. [62], which demonstrates no significant correlation between ACE or ACE2 expression with survival. Unfortunately, our limited sample size prevents us studying a similar correlation with survival. A recent pan-cancer bio-informatics study also identifies increased ACE2 expression in RCCC [67], with increased expression correlating with improved survival [67]. These findings are reflected by other studies using data from the TCGA, where downregulation of ACE2 in RCCC is associated with worse survival [68,69]. Further associations include tumor progression, response to immunotherapy, stemness, and endothelial-to-mesenchymal transition [68]. The role of ACE2 in diverting signaling away from the ACE/ATII/AT_1_R axis may explain its seeming protective role in malignancy. The cleavage product of ACE2, angiotensin(1–7), has been trialed in a phase I clinical trial as anti-angiogenic treatment for solid tumors [70]. In contrast, it has also been suggested that angiotensin(1–7) promotes malignant cell migration and invasion in RCC [71].

The role of AT_1_R has been previously investigated in RCC, with increased expression of both AT_1_R and AT_2_R being associated with increased aggressiveness and reduced progression-free survival [72]. Captopril, an ACEI, significantly reduces tumor development in a xenograft model, although the exact mechanism has not been elucidated [73]. Reduced tumor growth and metastasis [49] and inhibited tumor angiogenesis and metastasis [47] have been demonstrated with AT_1_R antagonism in murine models. Epidemiological studies have demonstrated that administration of RASIs is associated with increased survival in patients with metastatic RCC [46,74,75,76], with beneficial responses in a phase II clinical trial for advanced RCC [77].

Interestingly, AT_2_R, a G-coupled transmembrane protein, was demonstrated within both the cytoplasm and the nuclei of the CSCs by immunohistochemical and immunofluorescence staining. This suggests the presence of functionally distinct localizations of AT_2_R–as both a transmembrane protein and nuclear receptor [78,79]. It has been suggested that the nuclear location of AT_2_R may either amplify the actions initiated by membranous AT_2_R [80] or modulate activation of the membranous location, possibly through transcription regulation [78,79]. AT_2_R was detected weakly by WB, and it was not detected by RT-qPCR. This may be due to the possibility that the RT-qPCR primers used in this study may not fully cover all possible splice variants of AT_2_R [81]. Alternatively, mRNA degradation might have resulted in the lack of detectable AT_2_R mRNA. Low levels of AT_2_R detected in RCCC is also consistent with the proposed protective role of AT_2_R against cancer [40,42], although this remains a topic of further investigation.

Our finding of the presence of components of the RAS by the CSCs within RCCC is consistent with findings of our previous studies of buccal mucosal [22], lip [21] and oral tongue [20] SCC, glioblastoma [19], primary HNcSCC [23], and mHNcSCC [24], metastatic MM to the braPLin [25] and regional lymph nodes [26], and metastatic colon adenocarcinoma to the liver [27]. We propose CSCs may be a potential novel therapeutic target by manipulation of the RAS [82,83]. Further study with a larger sample size to enable subset analysis, and functional investigations, are needed to determine the precise role of the RAS in CSCs in RCCC, and the therapeutic potential of RASIs in the treatment of this aggressive cancer.

## Figures and Tables

**Table 1:** Demographic details of the 15 patients with renal clear cell carcinoma.

Gender	
Male	7 (46.7%)
Female	8 (53.3%)
Mean age (range)	66.6 (36.6–87.5) years
ISUP Grade	
Grade 2	8 (53.3%)
Grade 3	7 (46.7%)
Tumor stage	
Stage pT1x	2 (13.3%)
Stage pT1a	5 (33.3%)
Stage pT1b	1 (6.7%)
Stage pT2a	2 (13.3%)
Stage pT3a	5 (33.3%)
Status	
Alive	11 (73.3%)
Deceased	4 (26.7%)

## Supplementary Materials

The following are available online at https://www.mdpi.com/article/10.3390/biom11040537/s1, Table S1: Additional demographic details of the 15 patients with renal clear cell carcinoma, Table S2: RT-qPCR and WB results summarized by patient, Table S3: immunohistochemical staining results summarized by patient, Figure S1: Hematoxylin and eosin stained sections and controls for immunohistochemical staining with normal vasculature staining for ACE, Figure S2: Tissue negative controls for immunohistochemical staining, Figure S3: Controls for double immunohistochemical staining, Figure S4: Split images and negative control for immunofluorescence staining, Figure S5: Gel electrophoresis of RT-qPCR products, Figure S6: Subset analysis of RT-qPCR data, Figure S7: Full-length WB images.
