# Supplementary material for: Expression of Components of the Renin-Angiotensin System by Cancer Stem Cells in Renal Clear Cell Carcinoma
Source: Biomolecules. 2021 Apr 7;11(4):537. doi: 10.3390/biom11040537 (PMC8067590; doi:10.3390/biom11040537)
Supplement: Supplementary file 1 [file biomolecules-11-00537-s001.pdf]

biomolecules

MDPI

Expression of Components of the Renin-Angiotensin System by Cancer Stem Cells in Renal Clear Cell Carcinoma

Table S1. Additional demographic details of the 15 patients and their renal clear cell carcinoma.

Patient	Sex	Age*	Status	Survival Time $^{\delta}$	TNM Staging	ISUP Grade	Used for RT-qPCR/WB
1	Female	77.8	Alive	5.1	$pT_{3a}N_1M_x$	Grade 2	Y
2	Male	67.8	Alive	4.9	$pT_{1a}N_xM_x$	Grade 2	Y
3	Female	70.2	Alive	4.8	$pT_{1a}N_0M_o$	Grade 3	N
4	Male	73.8	Alive	4.7	$pT_{1a}N_xM_x$	Grade 2	Y
5	Female	65.2	Deceased	0.6	$pT_{1a}N_xM_x$	Grade 2	Y
6	Female	68.5	Alive	4.5	$pT_{1b}N_0M_0$	Grade 2	N
7	Female	87.5	Deceased	1.5	$pT_{3a}N_0M_0$	Grade 3	N
8	Male	36.6	Alive	4.3	pT_{2a} N_0M_0	Grade 2	N
9	Female	76.2	Alive	3.9	$pT_{3a}N_0M_0$	Grade 3	N
10	Male	42.5	Deceased	0.7	$pT_{3a}N_1M_0$	Grade 3	N
11	Female	62.6	Alive	3.8	$pT_1N_0M_0$	Grade 3	N
12	Male	59.2	Alive	3.7	$pT_{1a}N_xM_x$	Grade 3	Y
13	Male	67.9	Alive	3.7	$pT_{1a}N_xM_x$	Grade 2	N
14	Female	69.4	Alive	3.5	$pT_{2a}N_0M_0$	Grade 2	Y
15	Male	73.8	Deceased	1.8	$pT_{3a}N_xM_x$	Grade 3	N

*years; $^{\delta}$ survival following diagnosis (years); RT-qPCR, reverse transcription quantitative polymerase chain reaction; WB, western blotting; Y, yes; N, no.

Table S2. RT-qPCR and western blot results on snap-frozen renal clear cell carcinoma tissues from 6 patients.

Patient	RT-qPCR*						Western blotting			
	Renin	PRR	ACE	ACE2	AT_1R	AT_2R	PRR	ACE	ACE2	AT_2R
1	37.36	0.18	0.72	0.91	0.94	-$^{\delta}$	+	+	+	+
2	0.81	0.51	1.99	0.19	-$^{\delta}$	-$^{\delta}$	+	+	-	-
4	-$^{\delta}$	0.08	0.14	0.05	-$^{\delta}$	-$^{\delta}$	+	+	+	-
5	113.77	0.74	1.89	1.25	4.43	-$^{\delta}$	+	+	+	+
12	0.46	4.99	1.35	0.01	-$^{\delta}$	-$^{\delta}$	+	-	-	+
14	325.53	1.17	2.5	4.92	3.13	-$^{\delta}$	+	+	+	+

*results presented as $2^{\Delta\Delta CT}$ fold-change values; $^{\delta}$ not detected (sample had not reached the threshold by cycle 38); RT-qPCR, reverse transcription quantitative polymerase chain reaction.

Table S3. Immunohistochemical staining patterns of components of the renin angiotensin system in 15 renal clear cell carcinoma tissue samples.

Patient	Renin		PRR		ACE			ACE2		AT_2R	
	Cytoplasm	Nuclei	Cytoplasm	Nuclei	Cytoplasm	Nuclei	Endothelium	Cytoplasm	Nuclei	Cytoplasm	Nuclei
1	+	-	+/++	+	-	-	++	++	-	++	++
2	-	-	-	-	-	-	++	+/++	-	++	++
3	-	-	+	-	-	-	++	++	-	++	+
4	+/++	-	+/++	-	-	-	++	++/+++	-	+	-
5	++	-	+	-	-	-	++	+/++	-	++	++
6	+	-	+/++	+	-	-	++	+/++	-	++	++
7	++	-	+	-	-	-	++	+++	-	++	++
8	+	-	-	-	-	-	++	++	-	++	++
9	-	-	+/++	-	+	-	++	++	-	++	++
10	++		+	-	-	-	++	++	-	++	-
11	+	-	+	-	-	-	++	-	-	++	+++
12	+++	-	+++	+	-	-	+	-	-	++	+
13	+	-	++	+	+	-	++	++/+++	-	++	+
14	+/++	-	+	-	-	-	++	++/+++	-	++	++
15	-	-	++	-	-	-	++	+/++	-	+	+

Aggregate of positive staining	11/15	0/15	13/15	4/15	2/15	0/15	15/15	13/15	0/15	15/15	13/15

+, weak staining; ++, moderate staining; +++, strong staining; –, no staining.

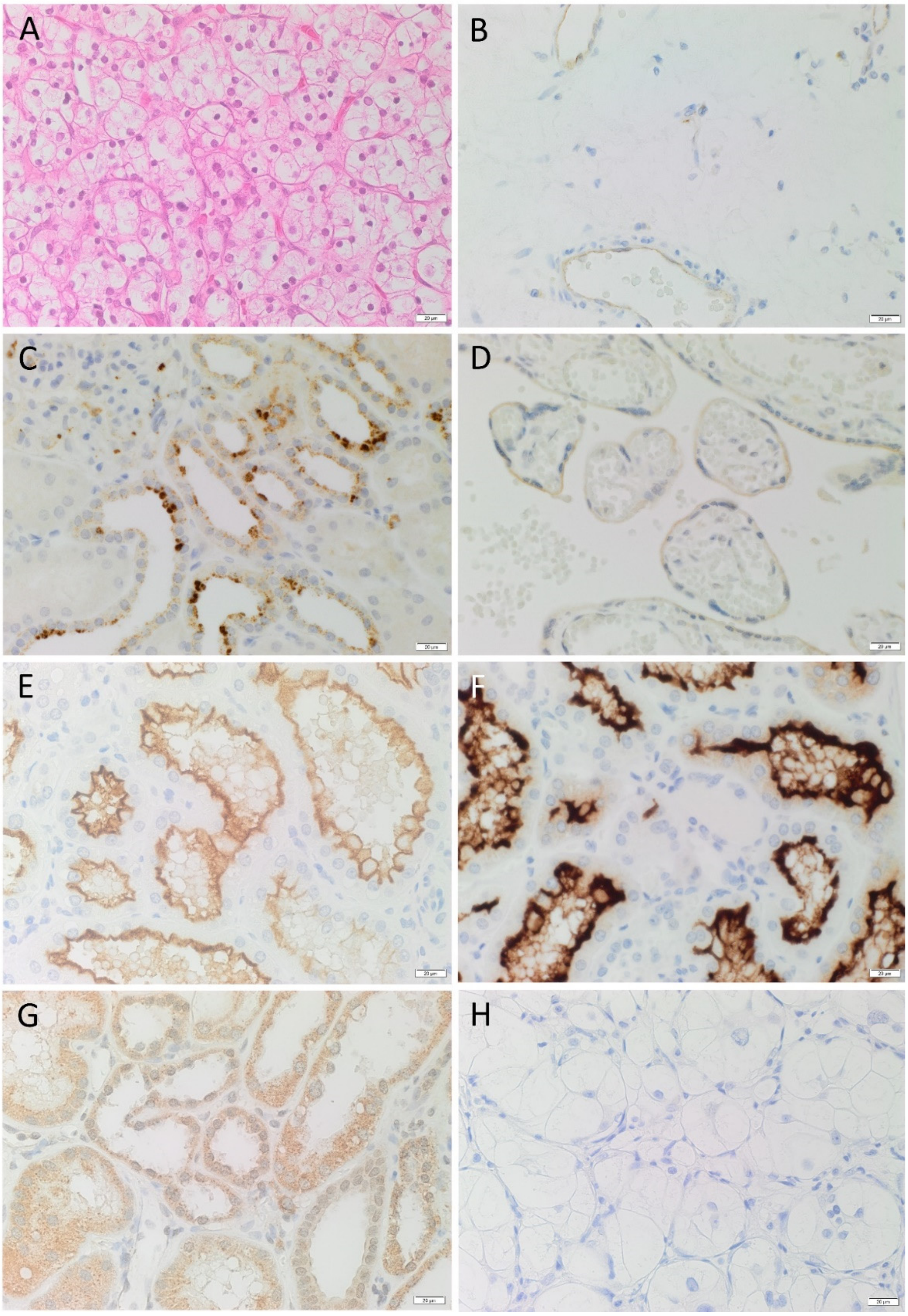

Figure S1. A representative hematoxylin and eosin-stained section of renal clear cell carcinoma (RCCC) tissue sample demonstrating the presence of the typical clear cells (**A**). Adjacent normal vasculature demonstrated staining for ACE (**B**, brown). Positive controls for immunohistochemical staining demonstrating the expected staining patterns on normal human kidney for renin (**C**, brown), placenta for PRR (**D**, brown), and kidney for ACE (**E**, brown), ACE2 (**F**, brown), and AT_2R (**G**, brown). A section of RCCC probed with matched anti-mouse isotype control (**H**, brown) confirmed the specificity of the secondary antibodies. Nuclei were counterstained with hematoxylin (**B**–**H**, blue). Original magnification: 400x. Scale bar: 20µm.

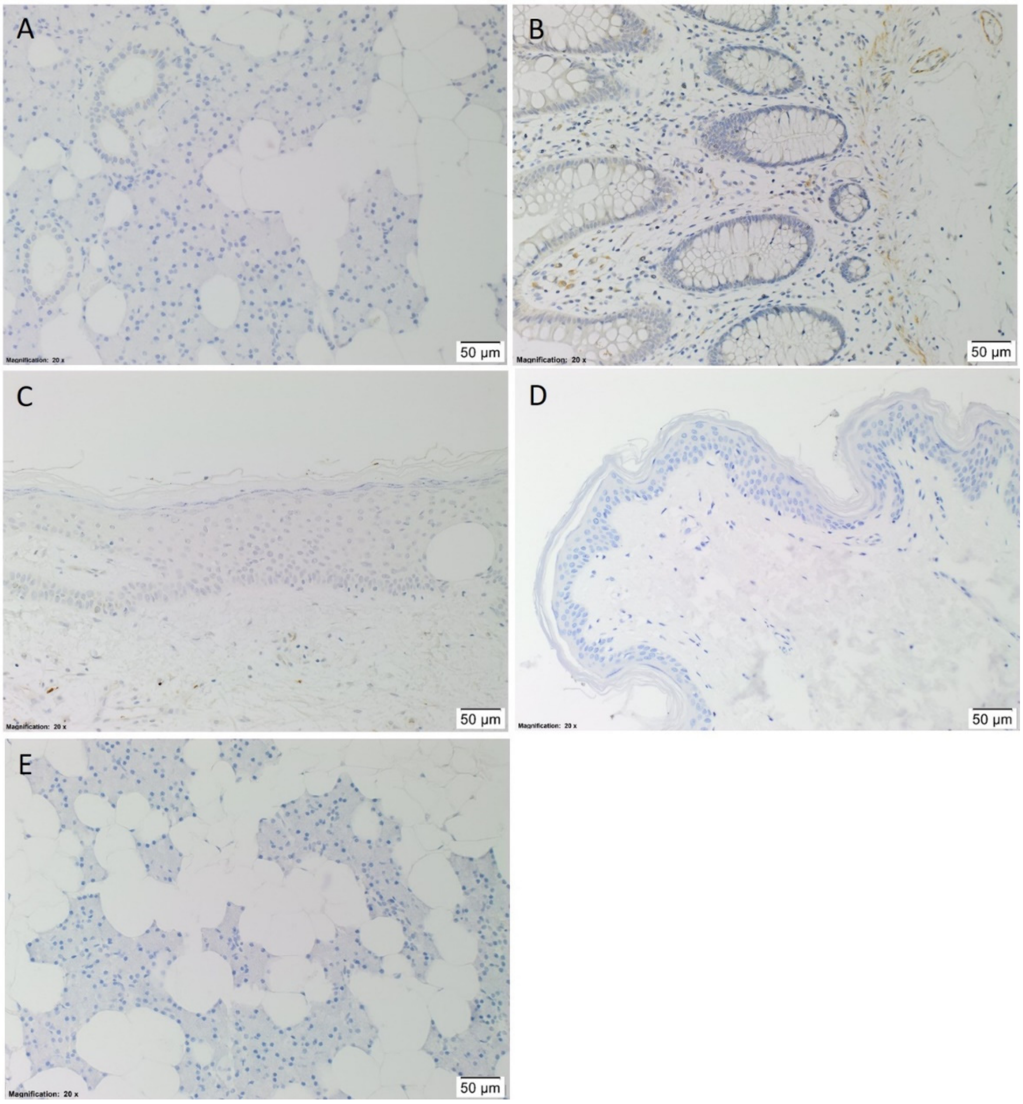

Figure S2. Tissue negative control images validating the specificity of primary antibodies used in immunohistochemical staining. Normal human tissues used were salivary gland for renin (**A**, brown), colon for PRR (**B**, brown), skin for ACE (**C**, brown) and ACE2 (**D**, brown), and salivary gland for AT_2R (**E**, brown). Nuclei were counterstained with hematoxylin (**A-E**, blue). Original magnification: 200x. Scale bar: 50µm.

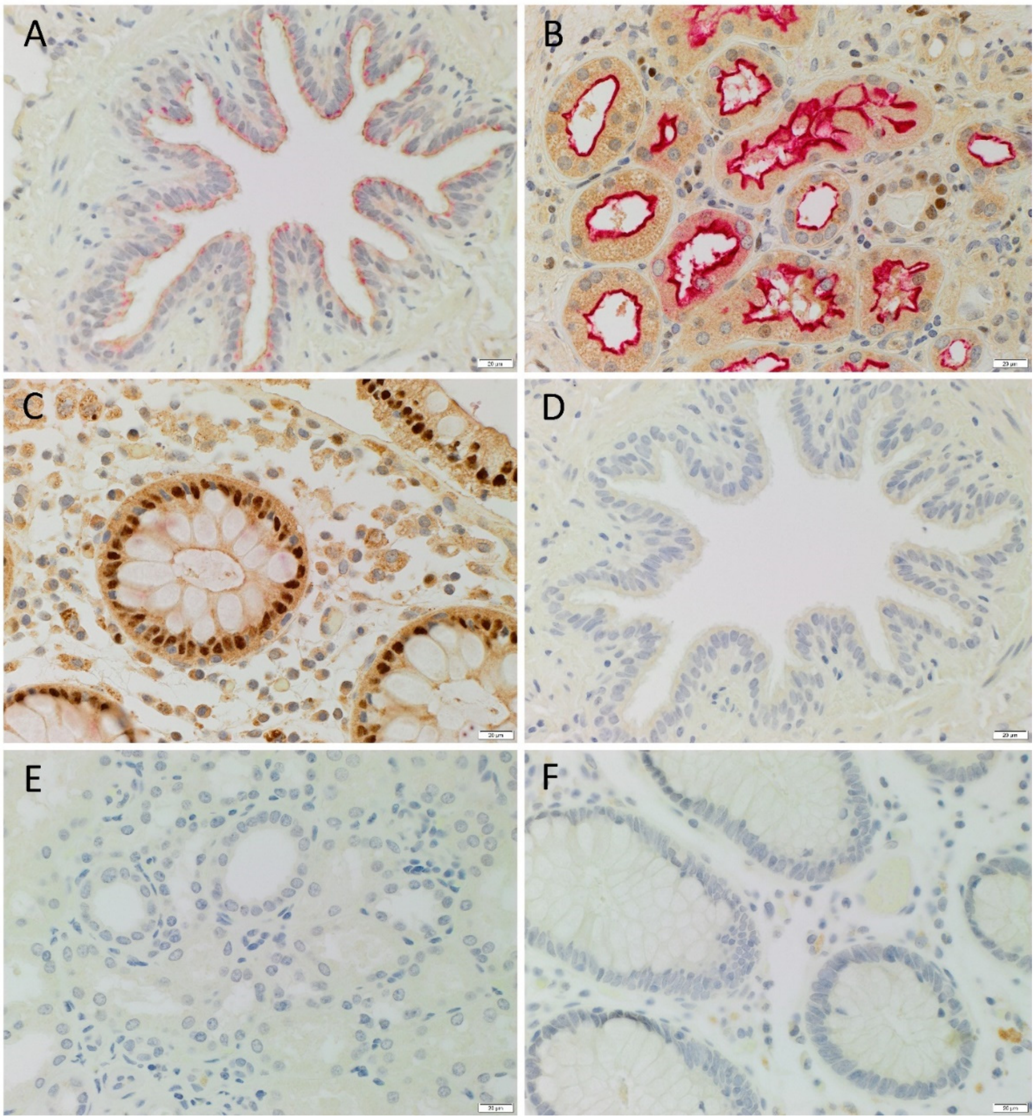

Figure 3. Positive and negative controls for double immunohistochemical stained renal clear cell carcinoma tissue sections. Normal human tissues for positive controls were bronchus for renin (**A**, red), kidney for ACE2 (**B**, red), and colon epithelium for KLF4 (**C,** brown). Isotype negative controls showed no staining in bronchus (**D,** red), kidney (**E,** red), or colon epithelium (**F,** brown). Nuclei were counterstained with hematoxylin (**A–F**, blue). Original magnification: 400x. Scale bar: 20μm.

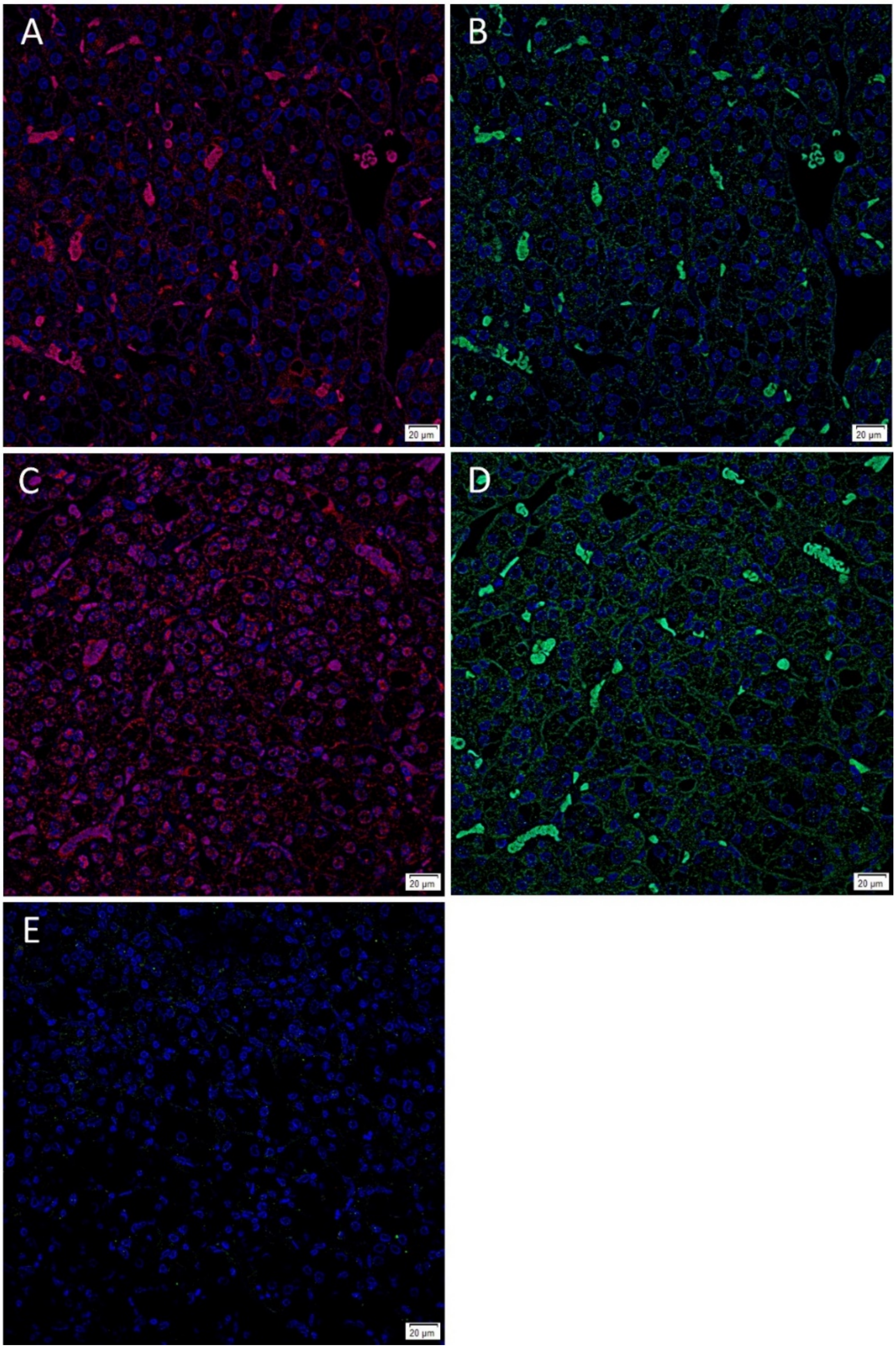

Figure S4. Split immunofluorescence-stained images of renal clear cell carcinoma shown in Figure 2 demonstrating expression of PRR (**A**, red) and OCT4 (**B**, green); AT_2R (**C**, red) and OCT4 (**D**, green); and isotype negative control (**E**). Cell nuclei were counterstained with 4′,6 diamidino-2-phenylindole (**A-E,** blue). Original magnification: 400x. Scale bar: 20μm.

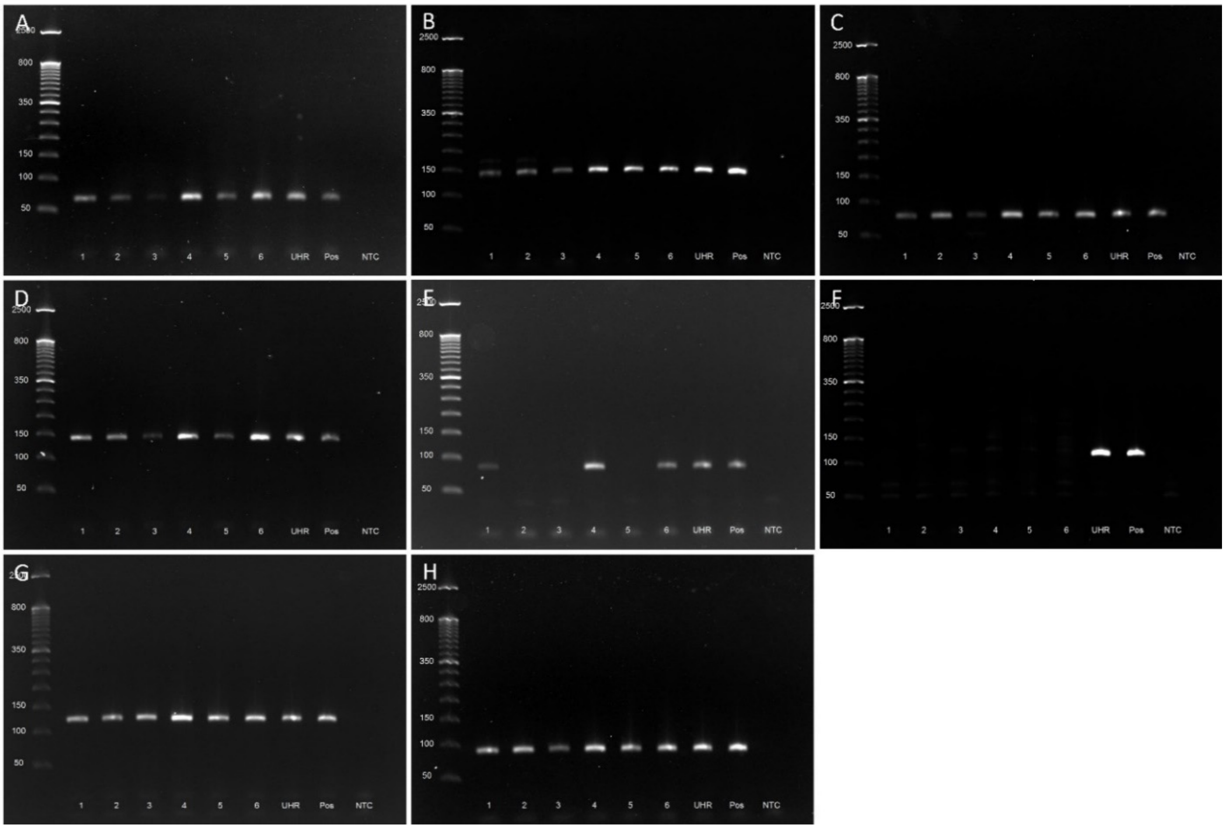

Figure S5. Reverse transcription quantitative polymerase chain reaction amplification products from six renal clear cell carcinoma (RCCC) tissue samples (**A**–**H**) were checked using agarose gel electrophoresis to confirm probe specificity for: renin (**A**, 62 bp), PRR (**B**, 141bp), ACE (**C**, 74bp), ACE2 (**D**, 141bp), AT$_1$R (**E**, 80bp), AT$_2$R (**F**, 113bp), GAPDH (**G**, 122bp) and PUM1 (**H**, 89bp). Ladder refers to the molecular weight DNA marker in base pairs (bp); Lanes 1-6 refer to the respective tissue samples; UHR, universal human reference RNA; Pos, positive control (RNA from uterine fibroid tissue, HepG2 cells, or PC3 cells according to the method); NTC, no template control (RNase-free water); No RT, No Reverse Transcriptase control. Only the expected size amplicon for each TaqMan assay was observed with no bands in the negative controls.

.

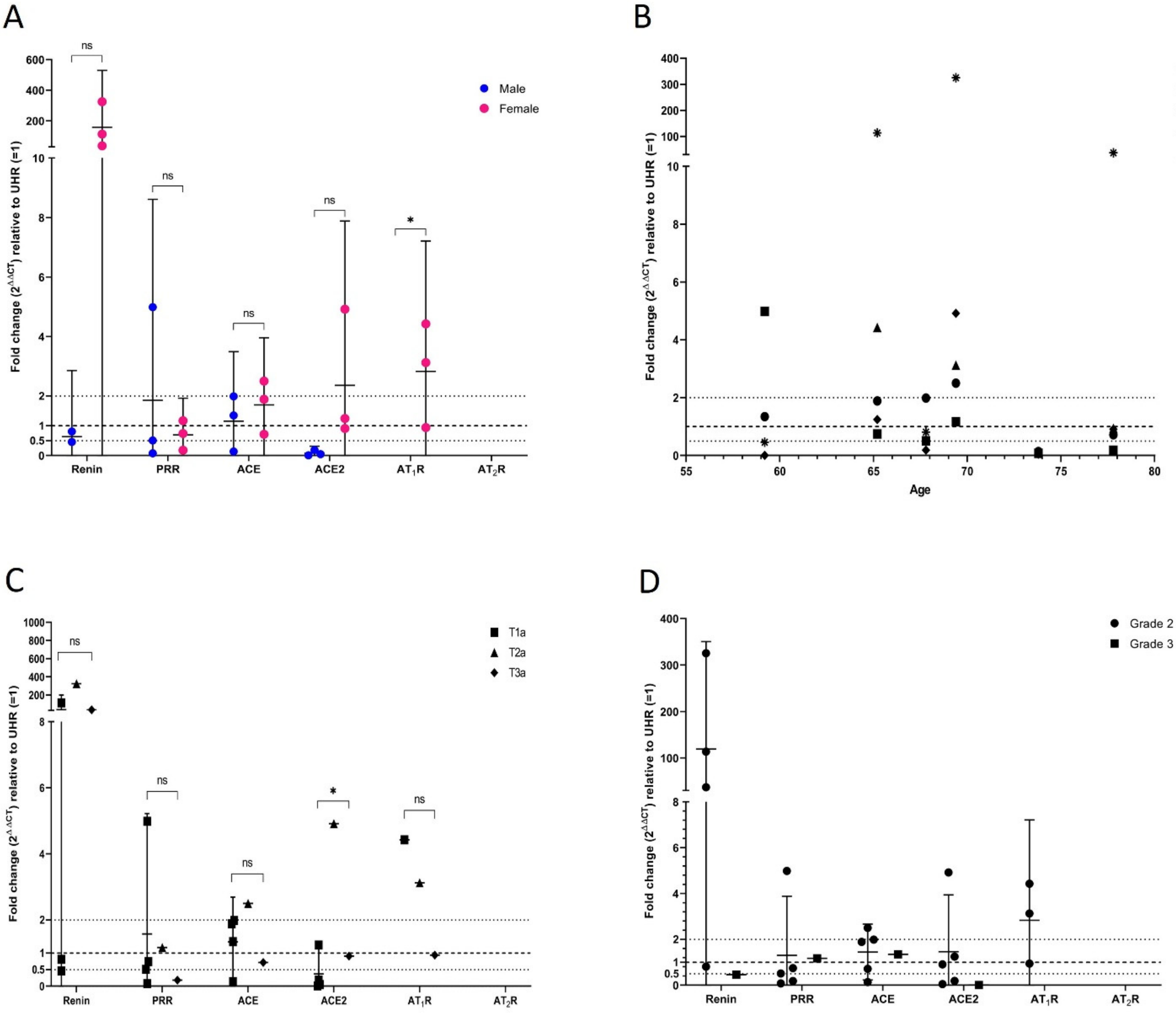

Figure S6. Subset analysis of RT-qPCR data as presented in Figure 4 by gender (**A**), age (**B**), tumor stage (**C**), and tumor grade (**D**). Statistical significance determined by un-paired t-test showed no significant differences found with age at diagnosis, and tumor grade. CT values were normalized to the reference genes GAPDH and PUM1, and displayed as expression relative to universal human reference RNA (UHR). Error bars represent 95% confidence intervals of the mean**.**

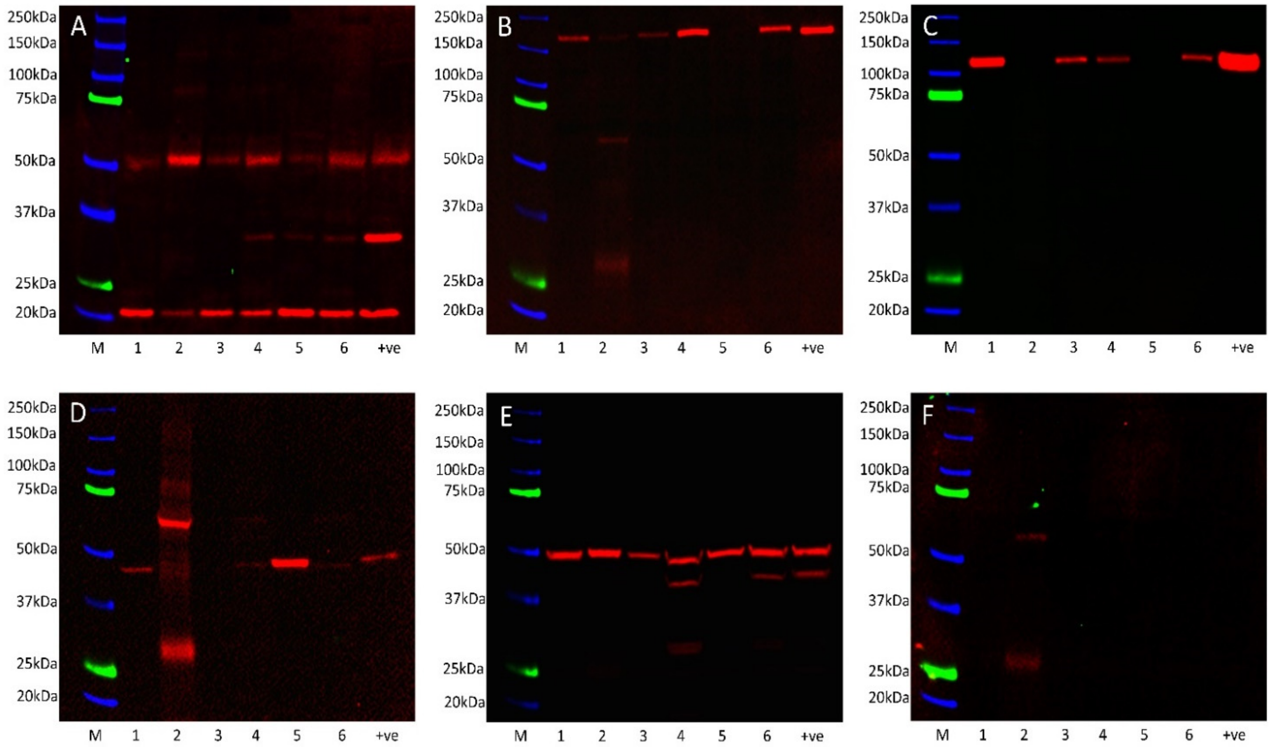

Figure S7. Representative full-length western blots presented in Figure 5. Full-length blots for PRR (**A,** red), ACE (**B,** red), ACE2 (**C,** red), and AT_2R (**D,** red). α-Tubulin confirmed similar total protein loading for each sample (**E,** red). Rabbit IgG isotype control (**F,** red) confirmed an instance of non-specific staining in the blot for AT_2R.
